# We Are What We Eat: A Stoichiometric and Ecometabolomic Study of Caterpillars Feeding on Two Pine Subspecies of *Pinus sylvestris*

**DOI:** 10.3390/ijms20010059

**Published:** 2018-12-24

**Authors:** Albert Rivas-Ubach, Josep Peñuelas, José Antonio Hódar, Michal Oravec, Ljiljana Paša-Tolić, Otmar Urban, Jordi Sardans

**Affiliations:** 1 Environmental Molecular Sciences Laboratory, Pacific Northwest National Laboratory, Richland, WA 99354, USA; Ljiljana.PasaTolic@pnnl.gov; 2 CREAF, Center for Ecological and Forestry Applications, Cerdanyola del Vallès, 08913 Catalonia, Spain; josep.penuelas@uab.cat (J.P.); j.sardans@creaf.uab.cat (J.S.); 3 CSIC, Global Ecology Unit CREAF-CEAB-CSIC-UAB, Cerdanyola del Vallès, 08913 Catalonia, Spain; 4 Grupo de Ecología Terrestre, Departamento de Biología Animal y Ecología, Facultad de Ciencias, Universidad de Granada, 18071 Granada, Spain; jhodar@ugr.es; 5 Global Change Research Institute, Czech Academy of Sciences, Bĕlidla 4a, CZ-603 00 Brno, Czech Republic; oravec.m@czechglobe.cz (M.O.); urban.o@czechglobe.cz (O.U.)

**Keywords:** plant-insect, metabolomics, stoichiometry, processionary moth, scots pine, secondary metabolites, herbivory

## Abstract

Many studies have addressed several plant-insect interaction topics at nutritional, molecular, physiological, and evolutionary levels. However, it is still unknown how flexible the metabolism and the nutritional content of specialist insect herbivores feeding on different closely related plants can be. We performed elemental, stoichiometric, and metabolomics analyses on leaves of two coexisting *Pinus sylvestris* subspecies and on their main insect herbivore; the caterpillar of the processionary moth (*Thaumetopoea pityocampa*). Caterpillars feeding on different pine subspecies had distinct overall metabolome structure, accounting for over 10% of the total variability. Although plants and insects have very divergent metabolomes, caterpillars showed certain resemblance to their plant-host metabolome. In addition, few plant-related secondary metabolites were found accumulated in caterpillar tissues which could potentially be used for self-defense. Caterpillars feeding on N and P richer needles had lower N and P tissue concentration and higher C:N and C:P ratios, suggesting that nutrient transfer is not necessarily linear through trophic levels and other plant-metabolic factors could be interfering. This exploratory study showed that little chemical differences between plant food sources can impact the overall metabolome of specialist insect herbivores. Significant nutritional shifts in herbivore tissues could lead to larger changes of the trophic web structure.

## 1. Introduction

Plants contain all the necessary nutrients for herbivore insects although the absolute amount can vary even within the same individual of a same plant species [1]. Most herbivore insects are specialized to feed on specific plant species or families whereas only a few are generalists, being able to feed on diverse plant species [2]. In this regard, several studies have proven the co-dependency between host plants and herbivores demonstrating a tight interdependent plant-insect co-evolution [3,4,5]. A balanced diet for an insect herbivore is critical for metabolic homeostasis, performance, and fitness [6,7,8]. Although insects present diverse mechanisms to regulate nutrient intake [1], the availability and nutritional content of food during larval development can directly influence the phenotype of adults [9]. A classic experiment demonstrated that caterpillars of *Heliothis zea* fed with diets with carbohydrates and proteins in a ratio of 1:4 showed the highest performance [10]. Later, Simpson et al. [11] showed that increases in insect performance is more related with the total abundance of carbohydrates and proteins in food rather than their proportions. In natural ecosystems, insects are often constrained in environments with non-optimal quality of nutrients [12] and natural selection could favor those herbivore insects able to regulate the nutrient intake at the needed proportions and concentrations [1].

The selection of foliage by insect herbivores has been widely discussed during the last decades. Commonly, foliar N and P concentrations have been identified as two of the main factors in foliar selection by insect herbivores; being more selected those plant individuals with higher nutrient abundance [13,14,15,16,17]. However, although plant selection based on specific nutritional requirements may occur in several insect species [18,19], it is still unclear if this is a general behavior [20,21,22,23]. Insect herbivores can regulate food intake to maintain a balanced diet for optimal fitness. Larval diet has proven to lead changes not only on larval performance but also on adult reproductive traits and body composition [24]. N concentration and digestibility of food are critical factors determining survival of Lepidoptera larvae, especially during early stages of larval development [18]. In fact, significant changes in larval performance and adult female fitness have been already reported in larvae feeding on different plant varieties of the same species [25]. It is known that insect-predators may include other nitrogen-rich predators in their diets, or even promote cannibalism [26], under situations of N limitation [27]. However, it is still unknown whether insect-prey selection by predators is actually driven by nutritional concentrations. Changes in plant nutritional concentrations could lead to cascade effects through trophic webs due to alteration of population dynamics (food abundance for predators) and the imbalance in nutritional intake by predators [16]. It is thus necessary to determine whether herbivores can maintain their elemental and metabolic homeostasis under changes in the plant source intake to further understand any potential impact on the structure of trophic webs.

Ecometabolomics, the study of the ecosystem structure and function through metabolomics analyses [28,29,30,31,32,33], has proven to be useful to understand the metabolic changes of organisms under the pressure of biotic and/or abiotic stressors [22,31,34,35,36,37,38]. The metabolome is defined as the total set of metabolites present in the organism at a particular abundance and moment [39]. The metabolome responds quickly to environmental fluctuations and stressors [28], and can be thus considered as the chemical phenotype of the organism [39,40,41]. Metabolomics analyses have demonstrated to be sensitive enough as to detect specific metabolic features upregulated or downregulated between different plant genotypes of the same species under herbivore attack [42], in understanding the success of an insect species feeding in different organs of a same plant [43,44] or even the impact of plant chemical defenses on insect folivores [45]. Metabolomics should thus allow the detection of any alleged metabolic shift in insect herbivores feeding on different plant species or varieties. This metabolomic information can, moreover, provide valuable information about the overall nutritional status of herbivores as most elements (i.e., C, N, P, …) do not act as themselves but as molecular compounds [46].

All the larvae stages of the pine processionary moth (hereafter; PPM), *Thaumetopoea pityocampa* (Denis & Schiffermüller), remain on the same host, being thus an excellent subject to study plant-insect relationships. Caterpillars of PPM are considered conifer-specialist herbivores since they are able to feed on several species of conifers [20,47,48]. The different stages of caterpillars of PPM elapse from end summer to beginning spring with a peak of folivory in winter [49]. The caterpillar of PPM is considered a pest and constitute a severe problem for many pine populations in the Mediterranean region [20]. Sierra Nevada Natural Park (South-East Spain) is a unique natural study site to examine whether specialist insects vary their overall metabolome structure and chemical composition when feeding on two different plant hosts. Two sympatric subspecies of *P. sylvestris*, the autochthonous *P. sylvestris subsp. nevadensis* (hereafter *nevadensis*) and the introduced *P. sylvestris subsp. iberica* (hereafter *iberica*), coexist together in some areas in Sierra Nevada Natural Park [50] since the mid-twentieth century and both subspecies are recently threatened by the PPM [18]. The main aim of this study is to examine whether caterpillars of PPM feeding on different plant hosts (subspecies of *P. sylvestris* (*nevadensis* and *iberica*)) present distinct metabolomes and nutritional properties. Although *nevadensis* and *iberica* belong to the same species, their overall metabolome structure and nutrient concentrations have proven to be significantly different [23,36]. We hypothesize that metabolomic differences between pine subspecies can lead to changes of insect herbivore metabolomes despite having physiological and metabolic mechanisms to regulate nutrient intake for maintaining body homeostasis. At the same time, we expect finding a direct relationship between overall nutrient concentration (C, N, P, etc.) in caterpillars and their plant hosts. We conducted liquid chromatography coupled to mass spectrometry (LC-MS)-based metabolomics, elemental, and stoichiometric analyses of caterpillars of PPM feeding on *nevadensis* (hereafter C-nevadensis) and on *iberica* (hereafter C-iberica), and leaves from attacked and non-attacked pines of each subspecies in Sierra Nevada Natural Park.

## 2. Results

The list of metabolites identified in caterpillars and pines are shown in Table 1. We identified 12 metabolites in pines which were not found in caterpillars; caryophyllene, sabinene, apigenin, catechin, catechol, epicatechin, epigallocatechin, kaempferol, luteolin, taxifolin, δ-tocopherol, and gibberellic acid 3.

Permutational multivariate ANOVA (PERMANOVA) on caterpillar metabolomes, nutrient, and stoichiometry data showed that herbivores feeding on different *P. sylvestris* subspecies have significantly distinct overall elemental, stoichiometric, and metabolome composition (*p* < 0.05) (Table 2). PERMANOVA accounted for about 10.3% of the overall variation between caterpillar groups (C-iberica and C-nevadensis) (Table 2).

One-way ANOVAs were performed for each individual variable to test for statistical significance between caterpillar groups. We found that 34.9% of the variables and a 32.1% of identified variables (elemental, stoichiometric and metabolomic features) changed significantly between caterpillar groups at *p* < 0.05. These percentages of significantly changing variables between caterpillar groups were 18.2% (all variables) and 9.6% (identified variables) for a significance level of *p* < 0.01. The list of metabolites showing marginal significant changes between caterpillars (*p* < 0.1) raised to 46.4% for the entire set of variables and 36.5% for the known variables (see Table S1 for univariate analyses of known variables).

**Figure 1:**
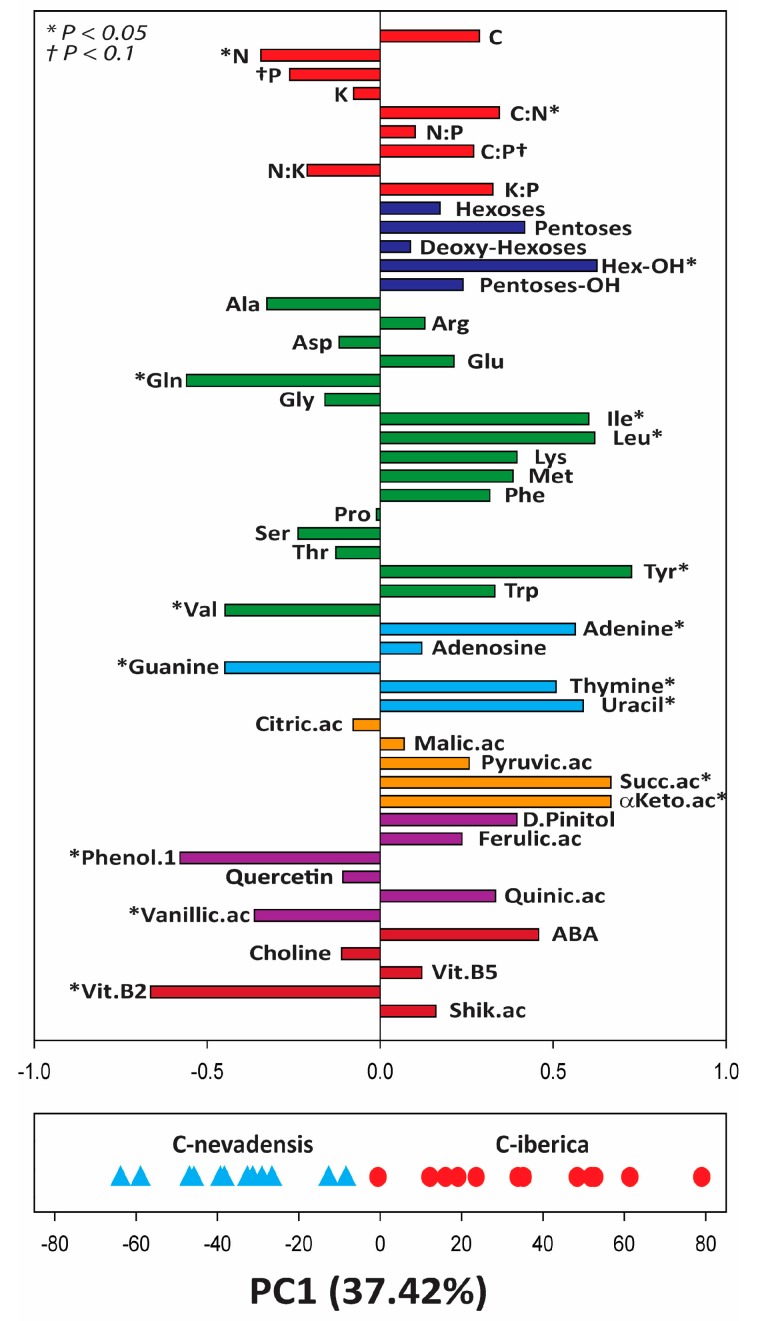
Variable and case plot of the principal component (PC) 1 of the principal component analysis (PCA) including all elemental, stoichiometric, and metabolomic data of the dataset of caterpillars (Caterpillar-Dataset). Only elemental, stoichiometric, and identified metabolomic variables are shown in the variable plot. Each bar represents the loading value for each variable on PC1. Elemental and stoichiometric variables are shown in red. Metabolite groups are shown in different colors: dark blue, saccharides (carbohydrates); green, amino acids; light blue, nucleobases/nucleosides; orange, organic acids related to tricarboxylic acid cycle; violet, phenolic compounds; and dark red, other. Caterpillars feeding on *Pinus sylvestris* ssp. *iberica* (C-iberica) are shown in blue triangles and caterpillars feeding on *Pinus sylvestris* ssp. *nevadensis* (C-nevadensis) are shown in red circles. Asterisks and crosses next to elemental, stoichiometric and metabolomic variables denote statistical significance (* *p* < 0.05) and marginal significance (^†^ *p* < 0.1), respectively.

Principal component analysis (PCA) performed with caterpillars alone showed clear separation between C-nevadensis and C-iberica along the principal component (PC) 1 (Figure 1). In general, the variable plot of the PCA showed that C-nevadensis had significantly higher N concentration and marginally higher P concentration than C-iberica. C-nevadensis had significantly lower C:N and marginally significant lower C:P ratios than C-iberica. The overall carbohydrate relative abundance was higher in C-iberica but only hexoses alcohol (hexoses-OH) showed statistical significance. Relative abundances of most amino acids did not change significantly between caterpillar groups with the exception of glutamine and valine that showed higher relative abundance in C-nevadensis and isoleucine, leucine, and tyrosine with higher abundance in C-iberica. The relative abundances of all identified nucleobases were significantly different between caterpillar groups; relative abundances of adenine, thymine, and uracil were higher in C-iberica while guanine was in relative higher abundance in C-nevadensis. Succinic acid and α-ketoglutaric acid had significantly higher relative abundance in C-iberica. We found several plant related compounds in caterpillar metabolomes; D-pinitol, ferulic acid, phenil-phenol, quinic acid, quercetin, vanillic acid, abscisic acid, and shikimic acid. Phenil-phenol and vanillic acid in higher relative abundance in C-nevadensis.

**Figure 2:**
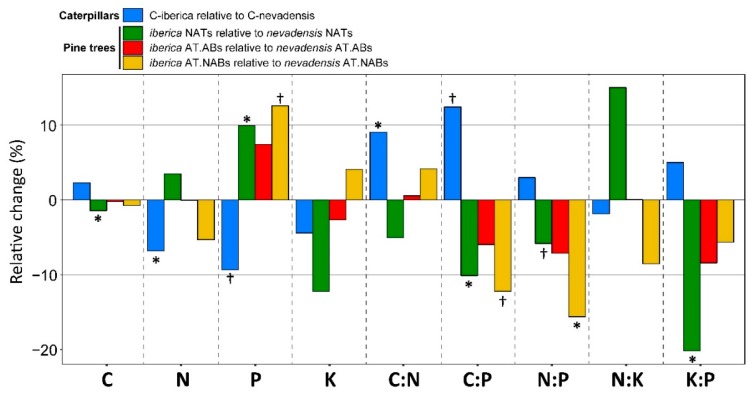
Relative difference (%) of elemental and stoichiometric variables between caterpillar groups (C-iberica relative to C-nevadensis) and between pine subspecies for each folivory level (*iberica* NATs relative to *nevadensis* NATs; *iberica* AT.ABs relative to *nevadensis* AT.ABs; *iberica* AT.NABs relative to *nevadensis* AT.NABs). Positive values indicate higher elemental concentration or stoichiometric ratio value in C-iberica or *iberica trees* while negative values indicate higher elemental concentration or stoichiometric ratio value in C-nevadensis or *nevadensis trees*. Asterisks and crosses denote statistical significance (* *p* < 0.05) and marginal significance (^†^ *p* < 0.1), respectively.

The relative differences (%) of elemental and stoichiometric variables of C-iberica respect to C-nevadensis were +1.4% for C (not significant; hereafter N.S.), −3.5% for N (*p* = 0.023), −9.3% for P (*p* = 0.072), +9.05% for C:N (*p* = 0.039), +12.43% for C:P (*p* = 0.076), +3% for N:P (N.S.), −1.87% for N:K (N.S.), and +5.01% for K:P (Figure 2). From the plant side, not-attacked trees (hereafter NATs) of *iberica* showed significantly higher P and lower C:P, and marginally significant lower N:P than NATs of *nevadensis*. Elements and stoichiometric variables did not differ significantly between attacked branches of attached trees (hereafter AT.ABs) of both subspecies while not-attacked branches of attacked trees (hereafter AT.NABs) of *iberica* had lower N:P and marginally lower C:P ratios and higher P concentration than AT.NABs of *nevadensis*.

**Figure 3:**
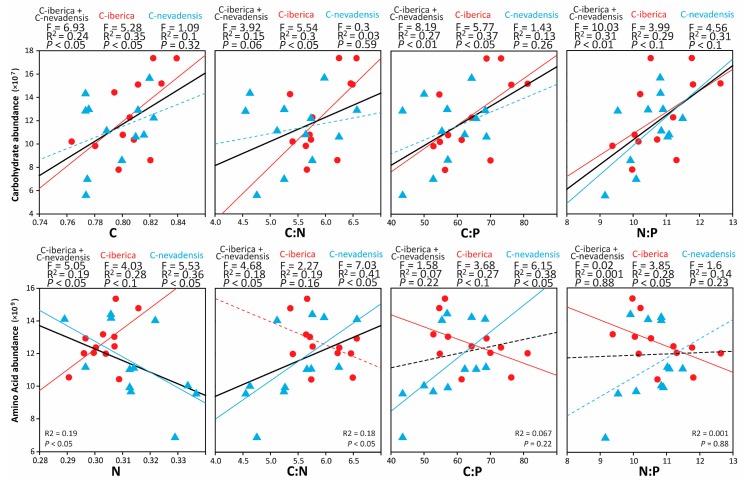
Pearson lineal correlations between total carbohydrate abundance versus C, C:N, C:P, and N:P ratios, and total amino acid abundance versus N, C:N, C:P, and N:P for both caterpillar groups (C-nevadensis and C-iberica) separately (*n* = 12) and together (*n* = 24). Coefficient of determination (*R*^2^), statistic Fisher value (F), and p value (*p*) are shown. Data corresponding to caterpillars feeding on *Pinus sylvestris* ssp. *iberica* (C-iberica) are shown in blue triangles and caterpillars feeding on *Pinus sylvestris* ssp. *nevadensis* (C-nevadensis) are shown in red circles. Lineal correlations for C-iberica and C-nevadensis are shown in blue and red, respectively. Lineal correlations for both C-iberica and C-nevadensis are shown in black. Correlations with *P* > 0.1 are represented by dashed lines.

Relationships between total carbohydrate abundance and amino acid abundance vs. C, N, C:N, C:P, and, N:P ratios in caterpillars were performed (Figure 3). C was correlated solely to total carbohydrate abundance and N was correlated solely to total amino acid abundance. Our results showed different tendencies between caterpillar groups; correlation carbohydrates vs. C, C:N, C:P, and N:P were positive and significant (marginally significant for C:N) when considering both caterpillar groups together. For C-iberica alone, we found significant positive correlations between total carbohydrates and C, C:N, and C:P and marginally positive correlation with N:P. However, we did not find significant correlation between total carbohydrates and C, C:N, and C:P in C-nevadensis, only N:P was marginally positively correlated with total carbohydrates (Figure 3). On the other hand, correlation between total amino acids and N was negative when considering caterpillar groups together and for C-nevadensis. Total amino acids vs. N correlation was marginally positively correlated in C-iberica. Total amino acids in C-iberica were negatively correlated with C:P (*p* < 0.1) and N:P and no significance were found with C:N. For C-nevadensis, we found positive correlation between total amino acids vs. C:N and C:P but no significance was found against N:P ratio (Figure 3).

**Figure 4:**
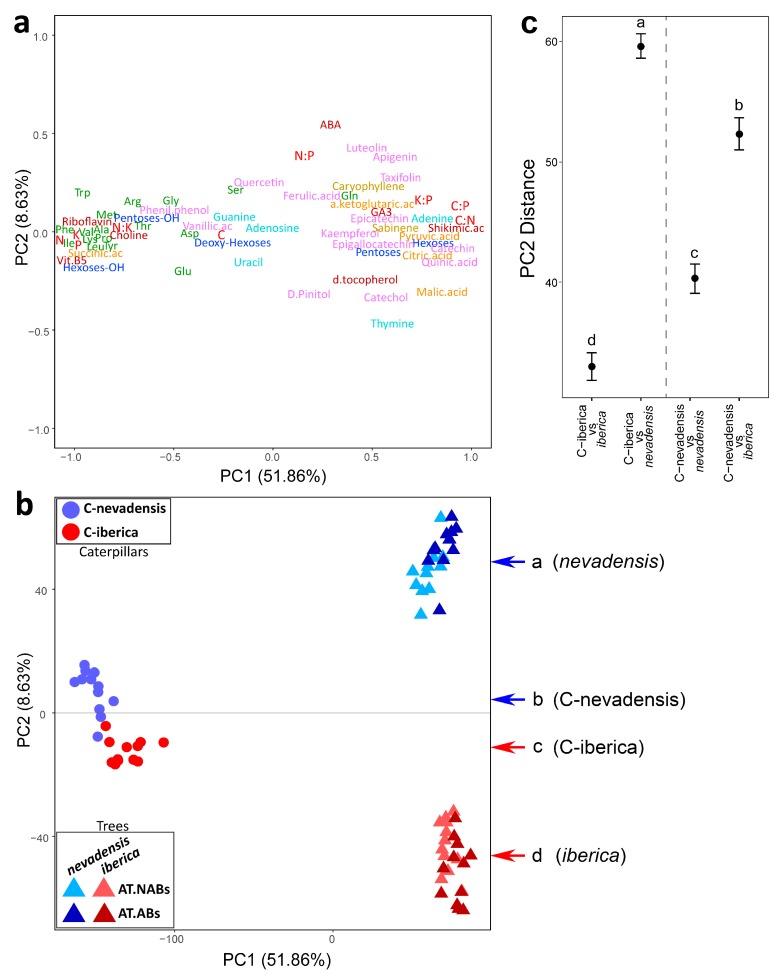
Variable (**a**) and case plot (**b**) of the principal component (PC) 1 vs. the PC2 of the principal component analysis (PCA) including all elemental, stoichiometric and metabolomic data of the dataset of caterpillars and pines (Pine-Caterpillar-Dataset). Only elemental, stoichiometric and identified metabolomic variables are shown in the variable plot (**a**). The location of a variable (element, stoichiometric ratio or metabolite) on the variable plot plane represents the loading value on PC1 vs. PC2 for such specific variable. Elemental and stoichiometric variables are shown in red. Metabolite groups are shown in different colors: dark blue, sugars (carbohydrates); green, amino acids; light blue, nucleobases/nucleosides; orange, organic acids related to tricarboxylic acid cycle; violet, phenolic compounds; dark red, other. Case plot (**b**) shows caterpillars feeding on *Pinus sylvestris* ssp. *iberica* (C-iberica) in blue circles and caterpillars feeding on *Pinus sylvestris* ssp. *nevadensis* (C-nevadensis) in red circles. Needle samples of attacked branches (AT.ABs) and non-attacked branches from attacked trees (AT.NABs) are shown in dark and light blue for *Pinus sylvestris* ssp. *Iberica,* and dark and light red for *Pinus sylvestris* ssp. *nevadensis*. Different letters next to each colored arrow denote significant difference between groups of samples after Tukey’s HSD post-hoc test (*p* < 0.05). One-way ANOVA plot (**c**) contrasting the metabolomic distances along the PC2 of the PCA between caterpillars and pines. Different letters indicate significant difference after Tukey’s HSD post-hoc test (*p* < 0.05). Not attacked trees are not represented in the PCA. Detailed discussion of the effects of the caterpillar on pines metabolism has been already published elsewhere [23,36].

The PCA performed including caterpillar and pines together showed clear clustering between caterpillars and pines along the PC1 (Figure 4b). In addition, the PC2 of the PCA clustered both pine subspecies (*nevadensis* and *iberica*) and caterpillar groups (C-nevadensis and C-iberica). Interestingly, C-nevadensis cases clustered closer to *nevadensis* pines than to *iberica* pines, and C-iberica cases clustered closer to *iberica* pines than to *nevadensis* along the PC2 (accounting for the 8.63% of the total variance) showing thus a certain degree of metabolic resemblance between plant hosts and herbivores. One-way ANOVA and Tukey’s HSD post-hoc test detected significant differences between all analyzed groups of samples along the PC2, being *nevadensis* and *iberica* the most distant groups of samples (Figure 4b). Caterpillars were also significantly clustered along the PC2 (F = 68.49; *p* < 0.0001). In addition, one-way ANOVA on the metabolomic distance between caterpillars and pines calculated along the PC2 of the PCA showed the “C-iberica vs. *iberica*” distance smaller than the “C-iberica vs. *nevadensis*” distance (Figure 4c). The same pattern was found for C-nevadensis; “C-nevadensis vs. *nevadensis*” distance was smaller than “C-nevadensis vs. *iberica*” distance. In general, caterpillar tissues had higher concentration of N, P, and K, together with higher N:K and lower C:N, C:P, and K:P ratios than pines. Few of the plant-related metabolites present in caterpillars (vanillic acid, and phenyl-phenol) were found at higher relative abundance than in plant tissues. In general, caterpillars had higher relative abundance of amino acids and lower abundance of phenolic compounds than pines.

## 3. Discussion

Our analyses found evidence supporting the hypothesis that caterpillars have different metabolomes and elemental composition when eating in different subspecies of *P. sylvestris* (Figure 1) and such difference accounted for over 10% of the total variability between caterpillar groups (Table 1). As previously shown [23,36], pine subspecies and different folivory levels (i.e., AT.ABs vs. AT.NABs) varied in their overall metabolomic structure. At the elemental level, we found C-iberica nutritionally poorer compared to C-nevadensis having higher C:N and C:P and lower total N and P concentrations (Figure 1 and Figure 2). However, the elemental and stoichiometric differences between caterpillar groups were not directly related to the nutritional concentrations of pines they fed on (Figure 2). In fact, NATs of *iberica* trees had slightly higher total concentration of P and lower C:P ratio compared to *nevadensis* suggesting that *iberica* trees could be more attractive than *nevadensis* from a nutritional point of view for oviposition by adult PPM females although unselective oviposition for PPM moths have been previously reported [18]. Nevertheless, once trees are attacked by PPM, the differences between subspecies in foliar P concentration and C:P ratios lost significance (*p* < 0.1 for AT.NABs and N.S. for AT.ATBs) (Figure 2) suggesting thus a significant adjustment of nutrients in *iberica* in response to folivory attack. This result clearly showed that nutrient transfer through trophic webs is not necessarily directly related with the nutritional concentration of food source and other factors such as the plant metabolome could play critical roles regulating nutrient transfer across trophic web levels. For example, folivory rates can change depending on nutrient concentration in food source, being those herbivores feeding on sources with high C:N and C:P ratios the most active to cope with nutrient demand [51]. The overall metabolomic differences between pine subspecies, as previously detailed and discussed elsewhere [23], could potentially cause nutritional imbalances in herbivores, forcing them to change their folivory rates to keep their nutritional requirements. Several lines of evidence proved that organismal and environmental C:N, N:P, and C:P ratios can play critical roles determining the ecosystem structure and function [14,52,53]. Therefore, significant differences in nutrient concentration between caterpillar groups (C-nevadensis with higher N, P concentrations and lower C:N and C:P ratios than C-iberica; Figure 2) could lead to significant changes in the population dynamics of the PPM, a species known for having episodic outbreaks depending on different factors such as food quality. The number of *nevadensis* trees in Sierra Nevada is recently much lower compared to *iberica* due the intensive felling during the twentieth century [20]. The replacement of autochthonous trees by *iberica* may cause significant changes in overall nutrient composition of PPM populations which could lead to shifts in population dynamics and consequently impact the trophic web structure [53,54,55].

Potassium (K) is an essential element for insects due to its high concentration in the hemolymph [56] and we did not find significant changes between caterpillar groups (Figure 1 and Figure 2). In plants, K is an important element to maintain a good performance of the physiological processes [57,58], especially at low water availability [57,59]. We found higher K concentration in caterpillars compared to pine needles (Figure 4) suggesting an accumulation of K in insect tissues, as expected with certain nutrients through trophic web levels. However, we found higher K:P and lower N:K ratios in pines compared to caterpillars (Figure 4) suggesting that the requirement of K in pines in proportion to other critical nutrients (N and P) is significantly higher than in caterpillars. This high difference is likely by the distant physiological functional traits between plants and insects. For example, K in trees play a critical function maintaining a large osmolite gradient from roots to leaves in order to sustain the cellular and physiological homeostasis [57]. Contrasting the elemental stoichiometry of plants vs. insects as shown here highlight the important role that K plays in plant physiology and should be considered in plant stoichiometric studies, especially under the pressure of stress conditions [29,57,59].

Among all the identified carbohydrates, only the group of hexoses-alcohol changed significantly between caterpillars (Figure 1). However, C-iberica had higher overall carbohydrate abundance than C-nevadensis (Figure 1) which could partly explain their higher tissue C:N and C:P ratios. Carbohydrates are C-rich compounds and, with exception of amino-sugar species, do not contain heteroatoms and are solely composed of C, H, and O [46]. Relationships of overall carbohydrate abundance vs. C concentration, C:N, and C:P ratios in caterpillars were significant when both groups were analyzed together (Figure 3) showing a significant body stoichiometry-metabolome relationship in insects, as previously reported in plants [29]. Such relationships were also significant for C-iberica considered alone but not for C-nevadensis, suggesting higher C allocation into carbohydrate metabolism in C-iberica; metabolic pathways generally related to rapid growth [29]. A general tendency in carbohydrate increases in relation to other molecular compounds could generate a nutritional imbalance in C-iberica and impact the trophic web structure by altering the population dynamics of PPM and the nutrient intake of their predators [26,27]. In addition, the overall increase of carbohydrates in C-iberica could be directly linked with the higher relative abundance of tricarboxylic acid cycle (TCA) organic acids (Figure 1). Higher abundances of TCA-related compounds and carbohydrates suggest higher respiratory metabolism in C-iberica with probably higher energy generation through the acetate oxidation directly derived from carbohydrates. However, our metabolomics results alone cannot definitely confirm this mechanism and additional analyses, i.e., proteomics, would help to decipher how those two groups of caterpillars assign C from carbohydrates to specific metabolic pathways. Interestingly, N:P body ratio did not significantly change between caterpillar groups (Figure 1 and Figure 2) but it was positively correlated with total carbohydrate abundance (Figure 3) even though the carbohydrates identified in our study do not contain heteroatoms. This result would be consistent with the expected in the frame of growth rate hypothesis when lower N and P concentrations and higher C:N and C:P ratios is expected to be related with higher N:P ratios [14,16].

Only a few amino acids changed significantly between caterpillar groups (Figure 1). However, we found that total abundance of amino acids was negatively correlated with N and positively with C:N body ratios of caterpillars when analyzing both caterpillar groups together and in C-nevadensis (Figure 3) suggesting that a large part of N concentration in C-nevadensis may be part of non-soluble structural proteins or other N-rich compounds such nucleotides as guanine and cytosine with five and three N atoms, respectively. Guanine is paired with cytosine and adenine with thymine/uracil and our analyses showed that C-nevadensis had higher relative abundance of N-rich nucleotide pairs (Guanine-Cytosine with eight atoms of N per pair) and lower relative abundance of adenine-thymine/uracil pairs (seven atoms of N per pair) than C-iberica. This result could partially explain the higher N concentration in C-nevadensis and suggests that the differences between *nevadensis* and *iberica* pines at both elemental and metabolomic levels, although similar still significantly different between them [36], could change the genome expression and protein synthesis in insect herbivores. From an evolutionary perspective, those changes could be selected trough several generations favoring different DNA pairs depending on the environmental (or food source in this case) N:P ratio as previously shown in plants under poor N availability [60].

Due to practical limitations in ecometabolomics research in general, and the complex logistics of this study, caterpillars were processed with the digestive content included. The dry weight of Lepidoptera larvae gut content can reach up to 20% of total caterpillar biomass when full [61]. We acknowledge this methodological limitation in our study which could lead to a bias in our results. However, the effects of temperature on feeding activity of the caterpillar of the PPM is well known and it only occurs when night temperatures are >0 °C and the temperature of the previous day was >6 °C [49,62,63]. Actually, some predictive models have been already developed to predict the feeding activity of the caterpillars of the PPM. For example, Battisti et al. (2005) [49] proposed a model to understand the feeding time of PPM caterpillars in areas of expanding range by using a threshold temperature of 0 °C combined with a more conservative activation temperature threshold of 9 °C, instead of 6 °C. According to the meteorological station at *La Cortijuela* (~1600 m a.s.l), night temperatures were below −5 °C for at least three consecutive days before caterpillar sampling and day temperatures did not reach +5 °C (see Material and Methods section). Therefore, caterpillars probably did not go out of their winter tents to feed for at least few days before they were sampled (4 March 2011). We even expect lower temperatures in the study site at *Collado the Matasverdes* as it is located over 300 m. a.s.l. from *La Cortijuela*. Given the non-optimal meteorological conditions for PPM caterpillars at the moment of sampling, we assume that caterpillars were already in a status of semi-starvation and the majority of the analyzed biomass corresponded to the insect and not to the content of guts. Furthermore, we also expect that most molecular components in the gut would have been substantially modified through the different digestion processes if not already assimilated or excreted by the caterpillars. We detected several plant-related compounds in the metabolome of caterpillars such as polyphenolics (Figure 1 and Table 1) which could be directly detected from the gut content, however, the relative abundance of compounds such as phenil-phenol and vanillic acid was higher in caterpillar tissues than in pines (Figure 4). If caterpillars of the PPM digested such compounds or had specific mechanisms to excrete them [64], the detected signal in larvae tissues would never be larger than in pine needles, the original source, thus suggesting bioaccumulation in insects [65]. It is known that insects have higher levels of certain vitamins than plants [66,67]. We found higher relative abundance of some vitamin-b complex compounds (choline, vitamin B5 (panthothenic acid), and vitamin B2 (riboflavin)) in caterpillars compared to pine needles (Figure 4). The requirement of vitamins for insects is well known, especially the B-complex group [68] which play important roles in cell metabolism. The relative abundance of riboflavin was higher in C-nevadensis compared to C-iberica, and in addition to its important role in the carbohydrate, lipid, and amino acid metabolism, it is also involved in the activation of defensive responses in plants [69,70] and animals [71] against biotic stresses (fungal, bacterial, and viral pathogens). Nevertheless, those responses are commonly species-specific and may vary depending on the infectious agent [70]. The potential sequestration of plant secondary metabolites (i.e., iridoids) by folivores to be used against parasitoids has been already suggested [72]. Glucosinolates are an example of defensive metabolites present in abundance in Brassicaceae plants that are accumulated and used by herbivores against parasitoids [73]. We also found significant higher accumulation of riboflavin and other compounds such as vanillic acid and phenil-phenol in C-nevadensis (Figure 1) that could be used by caterpillars for their own defense against parasitoids. Even so, further focused research is necessary to understand whether caterpillars of PPM accumulate and use specific plant secondary compounds against biotic stressors.

In summary, although animals have complex physiological mechanisms to regulate their metabolism and maintain homeostasis and fitness, caterpillars of the PPM feeding on distinct pine subspecies presented significantly distinct overall stoichiometry and metabolome structure accounting for over 10% of their total variability (Figure 1, Table 2). In addition, although plants and insects have very distinct metabolome structure, metabolomes of caterpillars presented a certain resemblance to the metabolome of the pine subspecies where they fed on (Figure 4b,c), suggesting thus that the overall insect herbivore composition is linked to the metabolome of their hosts. Nutrient transfer (i.e., N and P) trough trophic levels is not necessarily a net sum from the abundance in food sources. The metabolites where N and P are assigned to can play critical roles determining the elemental composition of higher trophic levels. From the ecological point of view, the stoichiometric and metabolomic shifts of caterpillars feeding on *iberica* pines could significantly impact the nutrient transfer along trophic webs by altering the PPM population dynamics and nutritional composition of larvae, causing structural and functional changes at higher trophic levels due to the larger abundance of *iberica* trees compared to the autochthonous *nevadensis*.

This exploratory study using elemental, stoichiometric and metabolomic data to understand the relationship between *Pinus syslvestris* and caterpillars of the processionary moth raised some questions in the field of plant-insect interactions research that warrant an accurate and focused investigation:

1. Do insect herbivores have the ability to accumulate specific plant secondary metabolites to repeal parasitoids? If this ability is found in multiple insect groups, is it consequence of common ancestry or is consequence of multiple independent acquisitions (phenotypic convergence)?
2. Why nutrient concentration in insect herbivore tissues is not necessarily correlated with the nutrient abundance of their food source? Are there molecular compounds in the food source that can potentially impact the nutrient transfer across different trophic levels by forcing changes in herbivore physiology?
3. How flexible are specialist insects feeding in diverse plant species? Which are the key plant molecular factors constraining the food diversity of specialist herbivores?

## 4. Material and Methods

### 4.1. Study Site

Sampling was conducted in early-March 2011 (late-winter) in Collado de Matasverdes (37.05° N, 3.27° W; 1900 m a.s.l.), one of the sites where the subspecies of P. *sylvestris nevadensis* and *iberica* coexist [46] in Sierra Nevada National Park (Granada, SE Spain). The climate is Mediterranean, with cold winters and hot summers with usually severe drought. The mean annual precipitation is 945 mm and the mean annual temperature is 9.8 °C. January is the coldest month with an average monthly minimum temperature of −0.1 °C and July is the warmest month with an average maximum temperature of 30.1 °C. Rainfall is concentrated mainly in spring and autumn [74]. The maximum temperatures recorded in the meteorological station of *La Cortijuela* (~1600 m a.s.l; 37.08° N, −3.47° W) for the sampling period (1–4 March 2011) were 13.9, 2.0, 2.9, and 2.9 °C, respectively. The minimum temperatures for the sampling period were −7.3, −7.3, −6.7, and −5.9 °C, respectively. Daily temperatures after 5 pm were always below 0 °C during the sampling period. Pines and caterpillars were collected on 2nd and 4th March, respectively.

### 4.2. Experiment Design and Sampling of Needles and Caterpillars

Twenty-four adult *iberica* and *nevadensis* trees, >5 m in height and >45 years old, were semi-randomly selected as study cases (total *n* = 48). Twelve of those trees of each subspecies had no signs of caterpillar attack (NATs), and the other 12 trees had caterpillars of PPM on the canopy, easily located by their winter tents (2–4 per tree). From each affected tree (*iberica* and *nevadensis*), one of the winter tents of PPM were removed with a pole, opened and 30–50 caterpillars were collected, pooled, packed in paper bags and quickly frozen in liquid nitrogen. According to previous studies, no difference in fitness has been observed between caterpillars feeding on *iberica* and *nevadensis* [18]. All sampled caterpillars corresponded to their final instar (5th instar) of their biological cycle (~5–6 cm. long). In addition, 60–100 needles were collected from the NATs, from the attacked branches of the attacked trees (AT.ABs; branches with caterpillar tents) and from the non-attacked branches of the attacked trees (AT.NABs; branches without caterpillar tents). All plant samples were also packed and rapidly frozen in liquid nitrogen. To be consistent and due to the significant physiological differences between sunlit and shade leaves [75], all needles collected from NATs, AT.ABs and AT.NABs were all oriented to the South (sunlit). Winter tents were also south-faced. All samples were collected during a narrow window of time (11:00 am to 3:00 pm) under constant environmental conditions to avoid differences caused by plant diurnal rhythms.

### 4.3. Caterpillar and Foliar Processing for Elemental and Metabolomics Analyses

Briefly, frozen caterpillars and pine needles were lyophilized and subsequently ground with a ball mill operating at 1600 rpm (~30 Hz) for 8 min. The fine homogeneous powder produced from caterpillars and pine needles was stored at −80 °C until the extraction of the metabolites for liquid chromatography coupled to mass spectrometry (LC-MS) analyses.

### 4.4. Elemental Analysis

C and N concentrations from caterpillar and pine needle samples were determined with a CHNS-O Elemental Analyzer (EuroVector, Milan, Italy). For each analysis, 1.4 mg of dried sample powder was used.

P and K concentrations from caterpillars and pine needles were determined by acid digestion extraction in a microwave reaction system under high temperature and pressure [76] and analyzed by an ICP-OES (Optic Emission Spectrometry with Inductively Coupled Plasma) (Perkin-Elmer Corporation, Norwalk, CT, USA). Briefly, 250 mg of dried sample powder were added into a Teflon tube. Each tube subsequently received 5 mL of nitric acid and 2 mL of H_2_O_2_ and samples were digested in a MARSXpress microwave reaction system (CEM, Mattheus, NC, USA). The digestions were transferred into 50-mL glass flasks, resuspended in nanopure water to a final volume of 50 mL and analyzed by ICP-OES (Perkin-Elmer Corporation).

### 4.5. Extraction of Metabolites for Liquid Chromatography-Mass Spectrometry (LC-MS) Analyses

Polar and semi-polar metabolites from caterpillars and pine needles were extracted as described elsewhere [77] with minor modifications. Briefly, two sets of 2 mL centrifuge tubes were labeled: set A was to perform the extraction and set B was to keep the extracts from set A. For each sample, 30 mg of caterpillar powder and 100 mg of pine powder were weighted into their corresponding tube of set A. Subsequently, one mL of MeOH/H_2_O (80:20) was added to each tube, vortexed for 15 min, sonicated at 24 °C for 5 min and centrifuged at 15,000× *g* for 5 min. Thus, 0.6 mL of the supernatant (extract) from each tube of set A was transferred to the corresponding tubes of set B. This procedure was performed twice to the tubes of set A to perform two extractions on the same sample. The tubes of set B were centrifuged at 15,000× *g* for 5 min and each extract was collected by crystal syringes, filtered through 0.22 µm pore syringe microfilters and transferred to a labeled HPLC vial. Vials with extracts were stored at −80 °C until the LC-MS analysis.

### 4.6. LC-MS Analysis

Separation of metabolites was performed by LC with a reversed-phase C18 Hypersil gold column (150 × 2.1 mm, 3 µ particle size; Thermo Scientific, Waltham, MA, USA) and an Ultimate 3000 HPLC system (Thermo Fisher Scientific/Dionex RSLC, Dionex, Waltham, Massachusetts, USA). Chromatography operated at constant temperature of 30 °C at a flow rate of 0.3 mL per minute. For each sample, 5 µL were injected. We used water (0.1% acetic acid) (A) and acetonitrile (B) as mobile phases. Both A and B were previously filtered and degassed for 10 min in an ultrasonic bath. The elution gradient was set to begin at 90% A (10% B), maintained for 5 min and then changed to 10% A (90% B) during the next 15 min, and held for 5 minutes. The elution gradient was thus gradually recovered linearly to the initial conditions (90% A; 10% B) over the next 5 min. The chromatographic column was washed and stabilized for 10 min before injecting the next sample.

LC was coupled to an LTQ Orbitrap XL high-resolution mass spectrometer (Thermo Fisher Scientific, Waltham, MA, USA) equipped with an HESI II (heated electrospray ionization, Thermo Fisher Scientific) source for mass spectrometry analyses. All samples were injected twice; once with the HESI operating in positive ionization mode (+H) and once in negative ionization mode (–H). The mass spectrometer operated in FTMS (Fourier Transform Mass Spectrometry) full-scan mode with high-mass resolution (60,000) and mass range acquisition of 50–1000 *m*/*z*. A caffeine standard was injected every 15 samples to monitor the resolution and sensitivity of the spectrometer. Experimental blank samples were analyzed every 10 samples for sample background determination.

### 4.7. Processing of LC-MS Chromatograms

Chromatograms of both positive and negative ionization modes were analyzed separately. RAW files from caterpillars and attacked trees were processed together. Metabolomes of NATs were not included in this study as have been already discussed in previous studies [23,36]. Solely the elemental composition and the C:N:P:K stoichiometry was used from NATs in this study. MZmine 2.14.2 was used to process the raw data files obtained from the mass spectrometer [78]. All chromatograms were baseline corrected, deconvoluted, retention time normalized, aligned, and metabolic features were assigned to specific metabolites (See Table S2 for full parameter details). Metabolites were automatically annotated to specific metabolites by matching the detected features with the total exact mass and retention time of our in-house library generated from the measurements of hundreds standards in positive and negative ionization modes. This metabolite assignation method corresponds, thus, to a second level of identification confidence [79]. Numerical datasets were exported and the different identified variables corresponding to the same molecular compound were summed to end up with a single variable per identified metabolite (see Rivas-Ubach et al. [23] for details). The numerical values of the variables of the datasets correspond to the deconvoluted peak areas of the chromatograms detected by the spectrometer which are directly proportional to the abundance of the metabolic feature. For this reason, we used the term *relative abundance* hereafter when referring to differences in the amount of metabolites between groups of samples.

### 4.8. Statistical Analyses

After processing the metabolomics RAW files with caterpillars and pines together, we coupled the nutrient, stoichiometry and metabolomics data into a single dataset and it was subsequently filtered through the following steps:

(1) All zero values of the dataset were replaced for missing data (NA).
(2) For each variable (metabolite feature, element and stoichiometric ratio), outlier values were determined for each *cell* individually. A *cell* corresponds to each group of samples defined by the combination of each factor and level. Therefore, our study is mainly composed by 6 *cells*: Caterpillars feeding on *iberica* (C-iberica), caterpillars feeding on *nevadensis* (C-nevadensis), AT.ABs of *iberica*, AT.NABs of *iberica*, AT.ABs of *nevadensis* and AT.NABs of *nevadensis.* Since only stoichiometric and elemental data was used from NATs of both pine subspecies, this dataset was filtered separately. Detected outliers were replaced for NA and were defined as:
  (1) Upper Outliers→value>Q75+2×IQRLower Outliers→value<Q25−2×IQR
  where Q75 represents the third quartile, Q25 represents the first quartile and IQR is the interquartile range (IQR = Q75–Q25) of each variable and cell.
(3) Variables with less than 60% of data within all *cells* were removed from the dataset.
(4) Variables with signal to noise lower than 15 (determined by the signal from blanks analyzed during the sequence) were removed from the dataset.

After the initial dataset filtering, two definitive datasets were generated for statistical analyses: Caterpillar-Dataset and Pine-Caterpillar-Dataset. 

The Caterpillar-Dataset included the elemental, stoichiometric and metabolomics data of caterpillars alone (C-iberica and C-nevadensis). This dataset consisted in a total of 9494 variables; 9 of them corresponded to elemental and stoichiometric variables (C, N, P, K, C:N, N:P, C:P, N:K, and K:P) and the remaining 9485 variables were metabolic features which 44 of them were identified by our in-house metabolite library (Table 1). Additionally, for the Caterpillar-Dataset, we generated two additional variables by summing all the peak areas corresponding to the identified sugars (total carbohydrate abundance) and amino acids (total amino acid abundance), separately. Those two variables were posteriorly correlated with elemental and stoichiometric variables (Figure 3).

The Pine-Caterpillar-Dataset included the data from the attacked-pines and caterpillars together consisting in the same 9 elemental and stoichiometric variables and a total of 9643 metabolomic features, 55 of them identified (Table 1). For this dataset, the values for each metabolomic feature of each sample of the Pine-Caterpillar-Dataset was scaled by the total intensity of its chromatogram to allow comparisons of the relative abundance of compounds between pines and caterpillars.

This study mainly focuses on the relationship between plant-insect stoichiometry and metabolome structure, therefore, the metabolomics datasets were processed considering exclusively the original individual food source PPM: the attacked trees. Therefore, the metabolomics data of NATs were not analyzed in this study and only elemental and stoichiometric data were slightly discussed. The results focused on the comparison of the metabolomic changes between pines attacked by the caterpillars of the PPM were already discussed in previous publications [23,36,80].

To test for significant differences between metabolomes and stoichiometry of caterpillars feeding on different pine subspecies, a PERMANOVA test was conducted on the Caterpillar-Dataset using the Euclidian distance with the caterpillar group as fixed factor and setting the number of permutations at 10,000 (Table 2). To represent the variability between C-iberica vs. C-nevadensis, the Caterpillar-Dataset was subjected to principal component analysis (PCA) (Figure 1), one of the most used ordination analyses in metabolomics studies [81,82,83]. Additionally, all elemental, stoichiometric and identified metabolomic feature were individually submitted to one-way ANOVAs to test for differences between caterpillar groups (Table S1). An additional PCA with the Pine-Caterpillar-Dataset was performed (Figure 4) to plot together caterpillars and pines in the same multidimensional space and detect trends between cases of caterpillars and pines along the first two PCs. The coordinates of pines and caterpillars along the PC2 were subjected to one-way ANOVA to test for significant clustering between groups of samples along the axis. Additionally, the metabolomics distances of each caterpillar sample (C-nevadensis and C-iberica) with each pine subspecies sample (*iberica* and *nevadensis*) along the PC2 were determined (“C-nevadensis—*nevadensis*” distance; “C-nevadensis—*iberica*” distance; “C-iberica—*nevadensis*” distance; “C-iberica—*iberica*” distance). We used the linear segment between the PC2 coordinate of each caterpillar and pine sample as the distance unit representing the metabolomics distances between caterpillars and pines for such axis. In total, 1152 distances along the PC2 were determined (12 caterpillar samples × 2 caterpillar groups × 24 pine samples (AT.ABs and AT.NABs) × 2 pine subspecies). All distances were subsequently submitted to one-way ANOVA, followed with a HSD Tukey’s post-hoc test to test for differences between them. 

All statistical analyses were performed in R [84]. One-way ANOVAs were performed with the function *aov* from the package “stats” [84]. HSD Tukey’s tests were performed with the HSD.test function of the package “agricolae” [85]. Linear correlations were performed using *rcorr* function from the package Hmisc [86]. The PERMANOVA analysis was conducted with *adonis* function in the package “vegan” [87]. PCAs were plotted using the *PCA* function from “FactoMineR” package [88] with the missing data from the dataset imputed using *imputePCA* function from package “missMDA” [89]. All graphs were first performed in R [84] and subsequently graphically treated by Adobe Illustrator CS6 (San José, CA, USA).

## Figures and Tables

**Table 1:** Identified molecular compounds in caterpillar tissues and pine needles by liquid chromatography coupled to mass spectrometry (LC-MS).

Compound Class	Compound Name	Averaged Relative Abundance (Deconvoluted Ion Chromatogram Peak Area)	
		Caterpillars	Pine Needles
Carbohydrates	Hexoses (Glucose, Fructose, Mannose, Galactose, …)	37,234,258	75,422,781
	Pentoses (Arabinose, Ribose, Xylose, …)	9,983,894	12,228,327
	Deoxy-Hexoses (Deoxy-glucose, Deoxy-galactose, Fucose, …)	592,288.1	52,9697.9
	Hexoses-OH (Sorbitol, Mannitol, …)	3,303,206	69,191.52
	Pentoses-OH (Xylitol, Arabitol, …)	64,255,464	29,779,498
Amino acids	Alanine	2.55 × 10^8^	14,910,060
	Arginine	73,078,590	10,791,686
	Aspartic acid	1,060,014	603,470.4
	Glutamic acid	21,812,174	9,902,287
	Glutamine	18,562,019	35,001,363
	Glycine	9,044,821	3,829,374
	Isoleucine	4.38 × 10^9^	4.98 × 10^8^
	Leucine	55,187,379	1,556,441
	Lysine	3.12 × 10^8^	5,959,588
	Methionine	1.76 × 10^8^	65,552,885
	Phenylalanine	2.24 × 10^9^	2 × 10^8^
	Proline	1.5 × 10^9^	53,596,908
	Serine	10,026,643	7,239,244
	Threonine	18,231,142	8,067,142
	Tyrosine	2.14 × 10^8^	24,445,586
	Tryptophan	2.03 × 10^9^	4.8 × 10^8^
	Valine	4.39 × 10^8^	47,193,674
Nucleobases	Adenine	1,422,770	41,186,385
	Guanine	428,695.9	281,113.5
	Thymine	2,393,343	19,099,857
	Uracil	5,393,886	3,712,866
Nucleosides	Adenosine	1,733,495	1,297,581
Krebs cycle related organic acids	Citric acid	4,984,835	76,318,569
	Malic acid	6,556,566	93,520,587
	Pyruvic ac	2,293,721	8,504,367
	Succinic ac	1.68 × 10^8^	740,971.5
	α-Ketoglutaric acid	37,118.36	235,423.5
Terpenes	Caryophyllene		13,334,699
	Sabinene		2,857,821
Phenolics	Apigenin		21,878,949
	Catechin		1.54 × 10^8^
	Catechol		39,448,216
	d-Pinitol	34,467,901	30,806,094
	Epicatechin		219,812.1
	Epigallocatechin		376,194.4
	Ferulic acid	754,250.3	972,992.9
	Kaempferol		1,199,232
	Luteolin		442,780.3
	Phenil-phenol	883,635.4	110,703.6
	Quercetin	1,089,097	805,365.7
	Quinic acid	52,693,061	4.26 × 10^8^
	Taxifolin		8,041,666
	Vanillic acid	917,952.3	457,177.3
Other	Abscisic acid	502,407.6	3,853,610
	Choline	2.44 × 10^8^	67,441,330
	δ-tocopherol		3,376,661
	Gibberellic acid 3		1,050,073
	Vitamin B5 (Panthotenic acid)	38,952,234	1,464,870
	Vitamin B2 (Riboflavin)	6,437,087	94,010.85
	Shikimic acid	26,206,952	1.27 × 10^8^

Averaged relative abundance of the identified metabolites in caterpillars and/or pines correspond to the average of the peak area of the deconvoluted ion chromatogram of each compound for each group of samples (caterpillars and pines) (see Section 4.7 of Material and Methods for more detailed information).

**Table 2:** Permutational multivariate analysis of variance (PERMANOVA) of the complete dataset of caterpillars (Caterpillar-Dataset) including the metabolomics and stoichiometric data with caterpillar group (C-iberica and C-nevadensis) as categorical independent factor.

Source of Variation	Degrees of Freedom	Sums of Squares	Mean Square	Pseudo-F	*R* ^2^	*p* Value
Caterpillar group	1	1.30 × 10^19^	1.30 × 10^19^	2.524	0.103	0.0223
Residuals	22	1.13 × 10^20^	5.14 × 10^18^		0.897	
Total	23	1.26 × 10^20^			1	

## Supplementary Materials

Supplementary materials can be found at http://www.mdpi.com/1422-0067/20/1/59/s1.
