# Supplementary material for: We Are What We Eat: A Stoichiometric and Ecometabolomic Study of Caterpillars Feeding on Two Pine Subspecies of Pinus sylvestris
Source: Int J Mol Sci. 2018 Dec 24;20(1):59. doi: 10.3390/ijms20010059 (PMC6337320; doi:10.3390/ijms20010059)
Supplement: Supplementary file 1 [file ijms-20-00059-s001.pdf]

SUPPORTING INFORMATION

Rivas-Ubach et al., 2018

Table S1. One way ANOVAS for each elemental, stoichiometric and assigned metabolomic variables with Caterpillar groun (C-iberica and C-nevadensis) as categorical factor. Table shows the average value ±SE, Fisher-F and P value.

	C-iberica		C-nevadensis			
	**Mean**	**SE**	**Mean**	**SE**	**F**	***P* value**
C (arcsin transformed)	0.8	0.01	0.79	0.01	1.81	0.191754
N (arcsin transformed)	0.3	0	0.31	0	5.85	0.024344
P	0.83	0.03	0.92	0.04	3.56	0.072312
K	2.1	0.07	2.2	0.06	1.13	0.299381
C.N	5.9	0.12	5.4	0.19	4.78	0.039642
N.P	10.7	0.27	10.4	0.2	0.84	0.368944
C.P	63.3	2.8	56.3	2.5	3.47	0.075767
N.K	4.3	0.12	4.4	0.08	0.34	0.567284
K.P	2.5	0.05	2.4	0.05	2.72	0.113597
Hexoses	38203653	1623410	36264864	3362260	0.27	0.608756
Pentoses	10729138	728894.2	9238651	1267198	1.04	0.319013
Deoxy-Hexoses	560678.7	49505.1	623897.5	73687.2	0.51	0.48386
Hexoses-Alcohol	3743549	252558.4	2862863	241199.1	6.36	0.01942
Pentoses-Alcohol	68250079	8085937	60260848	7066016	0.55	0.464753
Ala	2.23E+08	32863052	2.88E+08	40094107	1.58	0.222594
Arg	64674139	13135380	81483042	18294248	0.56	0.463358
Asp	1141874	207998	978153.9	194728.8	0.33	0.571386
Glu	22300549	2324406	21323799	1947722	0.1	0.750428
Gln	9660641	1540375	27463397	5165922	10.91	0.003243
Gly	7492530	1187328	10597113	2179039	1.57	0.224047
Ile	4.82E+09	2.97E+08	3.93E+09	1.97E+08	6.2	0.020845
Leu	67193942	8403615	43180816	7328968	4.64	0.042497
Lys	3.57E+08	44346427	2.67E+08	37944112	2.39	0.136747
Met	1.93E+08	11753085	1.59E+08	18526118	2.35	0.139355
Phe	2.34E+09	99266164	2.15E+09	1.09E+08	1.56	0.224316
Pro	1.51E+09	1.79E+08	1.49E+09	2.18E+08	0.01	0.923183
Ser	8511583	860724.7	11541703	1596265	2.79	0.108921
Thr	17396743	1934229	19065541	2589340	0.27	0.610773
Tyr	2.74E+08	18556595	1.54E+08	30111445	11.5	0.002624
Trp	2.11E+09	1.3E+08	1.95E+09	1.25E+08	0.76	0.393033
Val	3.6E+08	18345087	5.17E+08	58337007	6.59	0.017582
Adenine	2106093	530326.8	739447.6	165652.9	6.05	0.022231
Adenosine	1665703	288793.8	1801287	293495.1	0.11	0.745056
Guanine	257736.6	41538	599655.3	120256.6	7.22	0.013452
Thymine	3319494	606800.4	1467193	280491.1	7.68	0.011148
Uracil	6685906	1050430	4101867	619506.6	4.49	0.045623
Cittric.ac	4560756	686585.8	5408914	1093188	0.43	0.517982

Malic.ac	5828199	883830.3	7284934	1227101	0.93	0.345875
Pyruvic.ac	2519821	334795.5	2067620	317250.5	0.96	0.33754
Succinic.ac	1.95E+08	14931073	1.41E+08	8242660	9.94	0.004609
a.Ketoglutaric.ac	44322.9	5350.9	29913.8	4428.9	4.3	0.049944
D.Pinitol	37502174	3063336	31433629	4169645	1.38	0.25338
Ferulic.acid	885783.1	235385.9	622717.5	159293.9	0.86	0.364712
Phenol.1	302640.6	38727.9	1464630	373275.4	9.59	0.00527
Quercetin	1170729	178433.3	1007466	239923.5	0.3	0.590543
Quinic.acid	59890166	9440554	45495957	5758142	1.69	0.206486
Vanillic.acid	612726.1	81644.4	1223178	245667.8	5.56	0.027675
ABA	588689.2	97447.5	416126	72543.4	2.02	0.169495
Choline	2.45E+08	35398254	2.44E+08	27388835	0	0.97644
Vit.B5	39610704	2347010	38293763	3482114	0.1	0.756768
Vit.B2	3676967	657253	9197206	1375258	13.12	0.001511
Shikimic.acid	27577569	1808108	24836335	4352147	0.34	0.56671

Table S2. Description of the processes and parameters applied to LC-MS chromatograms with MZmine 2.14.2 to obtain the metabolomic fingerprintings of *Pinus sylvestris* and caterpillars of the processionary moth samples for both positive and negative ionization modes.

		(+H & -H) Chromatograms
**1**	**Baseline correction – RollingBall baseline corrector**	
	Chromatogram type	TIC
	Use m/z bins	No
	wm	10
	ws	8
**2**	**Mass detection** (exact Mass)	
	Noise level	1×10^5
**3**	**Chromatogram builder**	
	Minimum time span	0.05
	Min highest intensity	1×10^5
	m/z tolerance	0.0005 Da or 6ppm
**4**	**Smoothing**	
	Filter width	5
**5**	**Chromatogram deconvolution (local minimum search)**	
	Chromatographic threshold	30%
	Search minimum in RT range (min)	0.1
	Minimum relative height	5%
	Minimum absolute height	1×10^5
	Minimum ratio of peak top/edge	2
	Peak duration range	0-0.5 min
**6**	**Isotopic peaks grouper**	
	m/z tolerance	0.0005 Da or 6ppm
	RT tolerance	0.25
	Max charge	1
	Representative isotope	Most Intense
**7**	**Retention Time Normalizer**	
	m/z tolerance	0.0005 Da or 6ppm
	RT tolerance	0.25
	Min Standard intensity	1×10^6

**8**	**Chromatogram alignment (join alignment)**	
	m/z tolerance	0.0005 Da or 6ppm
	Weight for m/z	80
	RT tolerance	0.25
	Weight for RT	20
**9**	**Gap filling (Peak Finder)**	
	Intensity tolerance	30%
	m/z tolerance	0.0005 Da or 6ppm
	Retention time tolerance	0.2
	RT correction	Yes
**10**	**Metabolite Assignation**	
	m/z tolerance	0.0005 Da or 6ppm
	RT tolerance	0.25

RT, retention time; m/z, mass to charge ratio
